# Haem-assisted dityrosine-cross-linking of fibrinogen under non-thermal plasma exposure: one important mechanism of facilitated blood coagulation

**DOI:** 10.1038/srep26982

**Published:** 2016-05-27

**Authors:** Zhigang Ke, Qing Huang

**Affiliations:** 1 Key Laboratory of Ion Beam Bioengineering, Hefei Institutes of Physical Science, Chinese Academy of Sciences, Hefei 230031, China; 2 National Synchrotron Radiation Laboratory, University of Science & Technology of China, Hefei 230026, China

## Abstract

Although blood coagulation facilitated by non-thermal plasma has been reported several years ago, the insight to the involved mechanisms is still rather limited. In this work, we report our discovery of a new mechanism for the haem-promoted blood-coagulation caused by non-thermal plasma treatment. The reason for the haem role is due to that its oxidized form, namely, hematin, can promote the dityrosine cross-linking of fibrinogen, the most important coagulation protein, to form a membrane-like layer on the surface of the treated blood with plasma exposure. Both haem and non-thermal-plasma generated hydrogen peroxide are requisite for the cross-linking process. We confirmed that fibrinogen can coordinate with the haem iron to form a protein-haem complex which shows pseudo-peroxidase activity, and in the presence of hydrogen peroxide, the complex can induce the dityrosine formation between fibrinogen molecules, leading to the fibrin network necessary for the blood coagulation. Understanding of such an underlying mechanism can be useful to guide more efficient application of non-thermal plasma in the management of hemostasis, thrombosis and etc.

Blood coagulation is a physiological process which involves the coordinated activation of various blood components, such as platelets, proteins, cells and so on^1^. Under the circumstances of vessel damage, the coagulation system is triggered with the formation of the platelet plug which sticks to the injured surface. Tissue factors are then activated, promoting the protein-based coagulation cascade in which thrombin is generated^2^. The produced thrombin converts fibrinogen, an important serum protein with a concentration of 2–4 g/L, to fibrin, a three dimensional protein network which together with platelets forms a stable and permanent plug preventing further blood loss^3^,^4^,^5^.

However, not all bleeding can be stopped via the physiological coagulation cascade. Hemorrhage is the major cause of death for soldiers in battlefield and also for civilian patients with traumatic injury between the ages of 5 and 44 years^6^,^7^. Management of hemostasis is vital to surgical success because inadequate control of bleeding is associated with increased mortality rates and higher costs of care. Methods to staunch bleeding include applying pressure, suturing, using thermal-based energy devices, or using hemostatic agents^8^. A large variety of hemostatic products have been developed and marketed in the last few decades, including gelatin, collagen, oxidized regenerated cellulose, polysaccharide spheres, thrombin, fibrin sealant, zeolite, chitosan, starch-related products and so on^2^,^9^,^10^,^11^.

Although so many hemostatic methods have been developed, many of them fail to meet all the requirements for efficient blood coagulation. Therefore, the search for ideal methods still continues. An ideal hemostatic method should not only stop blood flow effectively and quickly but may also have the ability to sterilize^12^. For meeting these two requirements, non-thermal plasma treatment is now emerging as an ideal option, which does not only have the ability to assist blood coagulation^13^, but also can be bactericidal^14^. In 2006, Fridman *et al.* first reported the effectiveness of non-thermal plasma on blood coagulation, and they experimentally showed that Floating-Electrode Dielectric Barrier Discharge (FE-DBD), a category of non-thermal plasma, could significantly hasten the coagulation of a drop of blood drawn from a healthy donor and also the bleeding from the cut on human spleen^13^. Later on, the non-thermal plasma induced blood coagulation was also confirmed in an animal model, where fast coagulation and wound closure were observed for the incision of hairless Sprague-Dawley rat ear upon non-thermal plasma exposure^15^. Compared with thermal plasma which has been successfully applied for controlling bleeding several decades ago^16^, non-thermal plasma does not damage surrounding healthy tissues because it maintains bulk temperature as low as room temperature.

In spite of the ability of non-thermal plasma to coagulate blood has been tested both *in vitro* and *in vivo*, the understanding of the mechanism and the interactions of non-thermal plasma with serum components involved in blood coagulation remains rather limited, even with much effort by many researchers. It has been reported that a clot layer was formed on the blood surface exposed to non-thermal plasma treatment which plays an important role in plasma-assisted blood coagulation^17^,^18^,^19^. However, the mechanism of the layer formation or which plasma-produced active species contribute to the process is still elusive.

Herein, we report for the first time that under non-thermal plasma exposure, haem molecule, an essential cofactor in aerobic organism serum, can promote the layer formation and protein cross-linking on blood sample surface, which is due to intermolecular dityrosine formation between fibrinogen molecules. The mechanism for the cross-linking process was thereby carefully scrutinized. We found that fibrinogen can coordinate with the haem iron to form protein-haem complex which shows pseudo-peroxidase activity and catalyzes the dityrosine formation between fibrinogen molecules in the presence of plasma-generated hydrogen peroxide. The information obtained from this work is critical for unveiling the mechanism of non-thermal plasma-assisted blood coagulation and may also extend the application of non-thermal plasma technology in other related biomedical fields.

## Results

### Hematin assisted clot layer formation and protein cross-linking on blood sample surface under non-thermal plasma exposure

**Figure 1:**
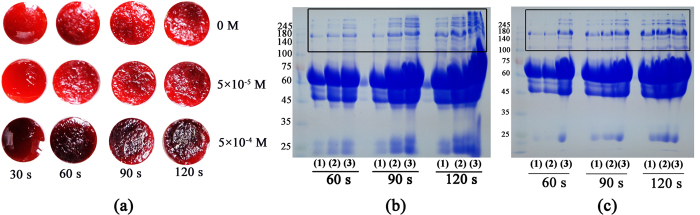
Effect of hematin on clot layer formation and protein cross-linking on blood sample surface. (**a**) Photograph of blood sample after non-thermal plasma exposure for 120 s in the absence/presence of hematin. Reducing SDS-PAGE analysis of dissolved clot layer formed on the surface of (**b**) blood and (**c**) blood plasma after non-thermal plasma exposure for 60, 90, and 120 s. The concentration of hematin in (1), (2), and (3) in panel (**b**,**c**) was 0, 5 × 10^−5^, and 5 × 10^−4^ M, respectively. Each experiment was repeated at least three times.

For discovering the effect of haem on blood coagulation under non-thermal plasma exposure, fresh pig blood in the absence/presence of hematin was exposed to non-thermal plasma for a period of 120 s. After treatment, a photo of the blood drop was taken immediately (Fig. 1a). Visually, a shell can be clearly seen on the blood sample surface with plasma treatment for 60 s and longer times. The presence of hematin clearly potentiate the gauffer of the formed layer especially at the concentration of 5 × 10^−4^ M with plasma treatment for 90 and 120 s. This result indicates that hematin promotes the clot layer formation on blood sample surface under non-thermal plasma exposure.

For further analysis, the layer with plasma treatment for 60, 90, and 120 s was picked up and dissolved in urea solution (6 M) and then separated by reducing SDS-PAGE (Fig. 1b). It can be seen that hematin promoted the formation of high molecular weight cross-linked protein polymers with molecular weights larger than 245 kDa which were stacked at the upside of 10% gel. Additionally, the blood-plasma (removal of red cells and platelets in the blood) obtained from fresh blood after centrifuging at 8000 rmp for 5 min was also treated by non-thermal plasma under the same conditions as mentioned above, and the formed layer was also analyzed by SDS-PAGE (Fig. 1c). Similar result was observed as shown in Fig. 1b, which confirms that hematin accelerates the cross-linking of the proteins inside the blood-plasma.

### Hematin assisted membrane-like layer formation and cross-linking of fibrinogen under non-thermal plasma exposure

**Figure 2:**
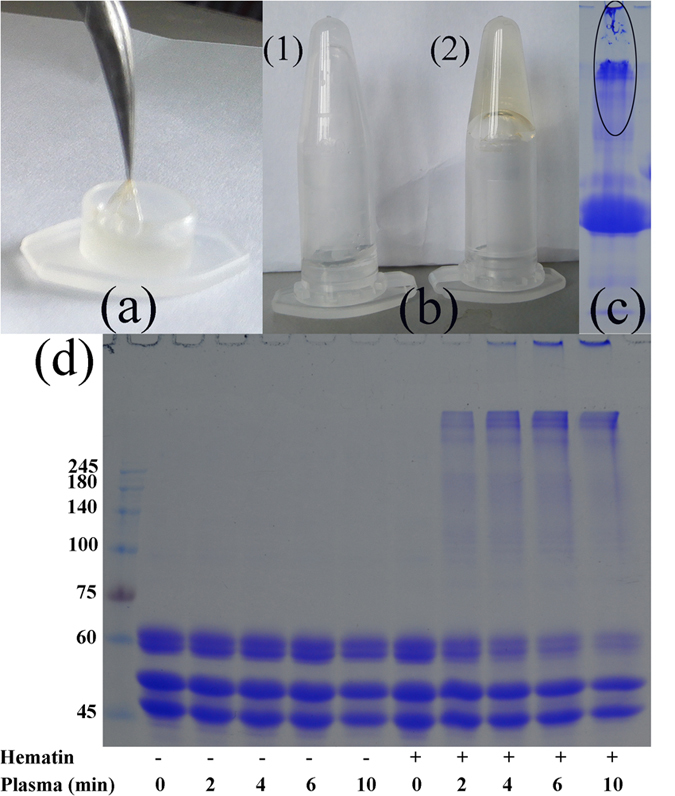
Hematin-assisted membrane-like layer formation and cross-linking of fibrinogen after non-thermal plasma exposure. (**a**) Photograph of the membrane-like layer formed on fibrinogen solution (10 mg/mL) surface in the presence of hematin (3 × 10^−5^ M) after non-thermal plasma treatment for 2 min. (**b**) Photograph of the fibrinogen solution (10 mg/mL) after non-thermal plasma treatment for 2 min in the absence (1) or presence (2) of hematin (3 × 10^−5^ M). (**c**) Reducing SDS-PAGE analysis of the layer in panel (**a)** dissolved in 6 M urea. (**d**) Reducing SDS-PAGE analysis of fibrinogen solution (2 mg/mL) in the absence/presence of hematin (5 × 10^−6^ M) after non-thermal plasma exposure. For the layer formation in panel a, the experiment was repeated three times and the photographs and SDS-PAGE patterns shown in panel (**b,c**) are the typical results. For panel (**c**) the experiment was repeated four times.

To find out whether the clot layer formation and protein cross-linking assisted by hematin under non-thermal plasma exposure (as shown in Fig. 1) was owing to the contribution of fibrinogen in blood, another set of experiments using pure protein fibrinogen were performed. Fibrinogen solution in 0.9% NaCl in the presence of hematin was also exposed to non-thermal plasma treatment, and we found that a membrane-like layer was produced on the solution surface (Fig. 2a). While if hematin was omitted, no obvious layer was seen. In another set of experiments, 0.5 mL fibrinogen solution in the absence or presence of hematin in 1.5 mL centrifugal tube was exposed to the same non-thermal plasma treatment for 2 min and then the tube was placed upside down. It can be seen that after plasma treatment the solution of fibrinogen in the presence of hematin was prevented to flow down while not occurred in the absence of hematin (Fig. 2b). The results shown in Fig. 2a,b therefore indicate that hematin can promote membrane-like layer formation on fibrinogen solution surface under non-thermal plasma exposure. Because of the requisite of hematin for the fibrinogen coagulation, the layer formation must be due to chemical reactions occurring in fibrinogen molecules induced by plasma exposure. If the layer was due to non-covalent aggregation of fibrinogen induced by physical factors produced by plasma such as heat, it would also be seen in the absence of hematin. To further investigate whether covalent cross-linking of fibrinogen occurred in the layer, it was collected and dissolved in urea solution (6 M) and then separated by reducing SDS-PAGE. As observed in Fig. 1b,c, high molecular weight cross-linked fibrinogen polymers larger than 245 kDa were formed and most polymers were stacked at the upside of a 10% gel. Moreover, some polymers could not enter the 4% stacking gel at all (the circle in Fig. 2c). Since no free sulfydryl exists in the fibrinogen molecule^20^, it can be excluded that the layer was due to disulfide bond formation.

Furthermore, we decreased the fibrinogen concentration to 2 mg/mL and exposed it to non-thermal plasma treatment in the absence/presence of hematin (5 × 10^−6^ M) in order to estimate how much of fibrinogen could be cross-linked at this concentration. Under this condition, the layer was diminished and the sample solution was separated by reducing SDS-PAGE. A similar PAGE pattern as with Fig. 1b,c, was observed (Fig. 2d). With the increasing of exposure time, an increase in the aggregates was observed at the expense of polypeptide chains. Most of the cross-linked polymers appeared very large with molecular weights larger than 245 kDa barely entering the matrix of 10% gel. Furthermore, with exposure time up to 6 min or 10 min, even some polymers could not enter the 4% stacking gel at all. The presence of hematin alone had no effect on the peptide cross-linking (Fig. 2d). When hematin was omitted from the sample, no cross-linking was observed even after plasma exposure for 10 min. Therefore, it can be concluded that hematin promotes the non-reducing cross-linking of fibrinogen molecules under non-thermal plasma exposure, and the cross-linked polymers eventually form a three-dimensional network leading to the membrane-like layer on the fibrinogen solution surface.

In addition, because there are other proteins such as albumin in the blood, it is also necessary to check whether other serum protein can also be cross-linked. For this purpose, other serum proteins including human serum albumin (HSA), hemoglobin, and γ-globulin were also exposed to non-thermal plasma in the presence of hematin. No membrane-like layer was formed on solution surface after plasma treatment and it failed to be cross-linked as it was examined with reducing SDS-PAGE analysis (Supplementary Fig. 1). Therefore, the results in Fig. 2 and Supplementary Fig. 1 unambiguously confirm that fibrinogen, in the presence of hematin, can be selectively cross-linked to form membrane-like layer on solution surface under non-thermal plasma exposure.

### The cross-linking and layer formation was due to dityrosine formation in fibrinogen molecules

**Figure 3:**
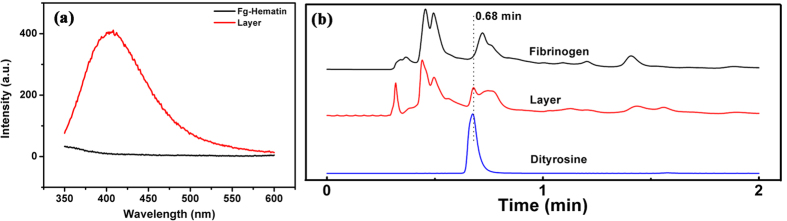
Dityrosine cross-linking in the membrane-like layer formed on fibrinogen solution surface in the presence of hematin after non-thermal plasma exposure. (**a**) Fluorescence spectra and (**b**) UPLC of the membrane-like layer formed on fibrinogen solution surface in the presence of hematin after non-thermal plasma exposure. Both experiments were repeated three times and two UPLC measurements were conducted for each sample. The figures in panel (**a,b**) are the typical results.

Considering the requisite of hematin for the fibrinogen cross-linking described above, it reminded us to associate this to the covalent cross-linking as occurring in β-amyloid peptides (Aβ) via dityrosine triggered by haem under oxidative stress^21^,^22^. To testify whether the cross-linking of fibrinogen was due to dityrosine in our case, fluorescence measurement of the layer in Fig. 2a dissolved in 6 M urea was conducted upon excitation at 330 nm. One prominent fluorescence band with the maximum emission wavelength at 410 nm was observed (Fig. 3a). This maximum emission wavelength is consistent with that of dityrosine^23^. In another experiment, the layer hydrolysate in 6 M HCl was separated by UPLC. The results illustrate the presence of a fluorescent peak eluting at 0.68 min coincident with authentic dityrosine (Fig. 3b), although its fluorescence intensity is relatively low.

Similarly, fibrinogen solution at 2 mg/mL after non-thermal plasma exposure was also analyzed with fluorescence emission spectroscopy (Supplementary Fig. 2). The fluorescence band of dityrosine at 410 nm was also observed as expected and the fluorescence intensity increased with prolonging the non-thermal plasma exposure time. Omission of either hematin or plasma exposure prevented the appearance of the fluorescent band, and the fluorescence band was not observed either for pure hematin solution after plasma exposure (data not shown). Additionally, selective modification of tyrosyl residues of fibrinogen was carried out using tyrosine-specific reagent *N*-acetylimidazole (NAI)^24^. Modified fibrinogen was then exposed to non-thermal plasma in the presence of hematin and analyzed with SDS-PAGE and fluorescence spectroscopy. It can be seen that chemical modification of tyrosyl residues inhibited the cross-linking of fibrinogen, and so suppressed the emission of the characteristic fluorescence band of dityrosine at 410 nm (Supplementary Fig. 3). Therefore, the results in Fig. 3, Supplementary Fig. 2, and Supplementary Fig. 3 have evidently proved that the membrane-like layer formation in Fig. 2 is due to the dityrosine cross-linking of fibrinogen molecules.

## Discussion

Fibrinogen is an important blood coagulation protein whose aggregation or cross-linking is a crucial step in coagulation cascade^3^. It is comprised of two identical subunits, each contains three polypeptide chains termed A*α*-, B*β*-, and *γ*, with molecular masses of 67, 55, and 48 kDa, respectively. All polypeptide chains are linked together by 29 disulfide bonds in a way that the six amino termini are held together in the central region. In response to vessel damage, soluble fibrinogens are cleaved to form fibrin monomers which are then aggregated to form three-dimensional fibrin network. The produced fibrin interacts with platelets, fibroblasts and endothelial cells to form a stable and permanent plug^4^,^5^. Based on the role of fibrinogen in coagulation, it has been widely applied as hemostat in surgery. Fibrin sealants consisting of concentrated fibrinogen and thrombin have been used to achieve hemostasis for nearly two decades in the United States^25^. Herein, we found that, in the presence of hematin, fibrinogen can be cross-linked via dityrosine formation to form a membrane-like layer on solution surface under non-thermal plasma exposure. The produced layer which has sufficient tensile force for supporting the mass of the solution (about 0.5 g) may play an important role in plasma-assisted blood coagulation. The cross-linking process is also the reason for the promoted clot layer formation and protein cross-linking on the surface of blood assisted by hematin under non-thermal plasma exposure as demonstrated by the experiment in Fig. 1.

Hematin is the oxidized form of haem which consists of an iron ion contained in the center of porphyrin and an essential prosthetic group in hemoglobin, the red pigment in blood. It is ubiquitous in blood, and in healthy individual blood its concentration is at about 0.2 μmol/L and can accumulate to 20 μM under hydrolysis^26^,^27^. Therefore, this mechanism of haem-assisted cross-linking of fibrinogen can be the major reason for the non-thermal plasma induced blood coagulation. In the foregoing experiments, however, oxidized haem (i.e., hematin in this work) was externally added into the blood and fibrinogen sample for verification or amplification of the coagulation effect. However, haem by itself is an essential component in blood. Also, because blood contains red blood cells which are rich in hemoglobin, under non-thermal plasma exposure, the red cells would be damaged and could also release haem into blood, thus possibly making an additional contribution to the haem-promoted coagulation process.

Therefore, to verify that fibrinogen in blood could be cross-linked without addition of external haem under non-thermal plasma exposure, we performed an additional two set of experiments. Firstly, the blood sample was treated by non-thermal plasma and it was found that free haem in blood was increased significantly (Supplementary Fig. 4). Secondly, fibrinogen solution (2 mg/mL) was mixed with 1/9 volume fresh blood and then the mixture was exposed to non-thermal plasma exposure. The fibrinogen solution mixed with 0.9% NaCl at the same volume ratio was used as the control. After plasma treatment, the sample was analyzed with reducing SDS-PAGE and the result is shown in Supplementary Fig. 5. It can be clearly seen that in the presence of blood the fibrinogen in the solution was also cross-linked after non-thermal plasma exposure (10 min). No protein aggregates were observed in the control group. Therefore, this experiment verifies that fibrinogen in blood can indeed be cross-linked under non-thermal plasma exposure even without addition of external hematin.

**Figure 4:**
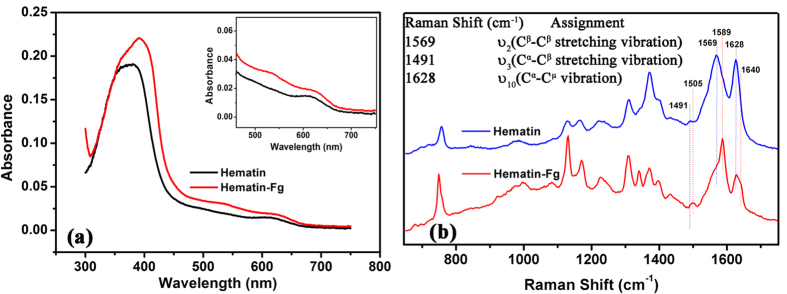
Fibrinogen-hematin formation. (**a**) UV-Vis absorption and (**b**) Raman spectra of free hematin and hematin-fibrinogen complex. The concentration of fibrinogen and hematin was 5 mg/mL and 2 × 10^−5^ M, respectively. Each experiment was repeated three times. For each sample, Raman measurements were conducted at 5 points at least.

Finally, the mechanism for the fibrinogen cross-linking process is worth further scrutinizing. It has been reported that Aβ can interact with haem to form five or six-coordinated complex which shows peroxidase activity and can lead to the formation of peptide oligomers containing dityrosine cross-linking in the presence of H_2_O_2_^21^,^22^. The observed cross-linking process in this work resembles the Aβ cross-linking reported previously^21^. To check whether fibrinogen coordinates with hematin to form complex, the UV-Vis absorbance and Raman spectra were measured and evaluated (Fig. 4). With fibrinogen added to hematin solution, the broad Soret band of hematin near λ = 385 nm is red-shifted to 396 nm, with additional visible bands at λ = 535 nm and the shoulder band at 410 nm (Fig. 4a), suggesting the formation of coordinated fibrinogen-hematin complex^21^. The vibrational frequencies in Raman spectra sensitive to the coordination number and spin state of hematin macromolecules are ν_10_, ν_2_, and ν_3_ and their assignment are listed in the inset of Fig. 4b^28–31^. The ν_2_ and ν_3_ bands of free hematin are observed at 1569 and 1491 cm^−1^, respectively. The depolarized spin state marker band at 1628 cm^−1^ indicates a six-coordinated high spin ferric species with an exchangeable weak field distal ligand^32^. After incubating with fibrinogen, ν_3_ and ν_2_ shifted to 1505, 1589, respectively, and a shoulder band at 1640 cm^−1^ (ν_10_) appeared, indicative of the formation of six-coordinate low spin protein-hematin complex^33^,^34^. The UV-Vis absorbance and Raman spectra indicate that coordinated fibrinogen-haem complex was indeed formed in our conditions.

As for the hematin-promoted cross-linking, it requires adequate H_2_O_2_ in the reaction system. Indeed, concentrations ranging from 50–350 μM of H_2_O_2_ were produced in solution by plasma treatment in our case (Supplementary Fig. 6). In order to evaluate the effect of plasma-generated H_2_O_2_ on the fibrinogen cross-linking, catalase serving as H_2_O_2_ scavenger was added to fibrinogen solution and then the sample mixture in the presence of hematin was exposed to non-thermal plasma. As shown in Supplementary Fig. 7, addition of catalase markedly inhibited the cross-linking of fibrinogen and the loss of polypeptide chains. This result illustrates the vital role of H_2_O_2_ in fibrinogen cross-linking and thus also confirms that the cross-linking process is a major reaction pathway for fibrinogen under non-thermal plasma exposure.

Therefore, we have confirmed that the mechanism of fibrinogen cross-linking via dityrosine formation triggered by haem molecule under non-thermal plasma exposure is similar to the case of Aβ peptides induced by haem molecule under H_2_O_2_ stress^21^. Coordinated fibrinogen-hematin complex shows peroxidase activity and in the presence of plasma-generated H_2_O_2_ the complex leads to the cross-linking of the peptides via dityrosine formation. Compared with the cross-linking of peptide via dityrosine formation induced by haem group under oxidative stress, non-thermal plasma produces active species in a local area such as the surface of solution and so the significant changes of fibrinogen occurs in this area while in bulk solution the biomolecules suffer minor changes. This is the reason why the membrane-like layer was formed on the solution surface described above. This nonhomogeneous reaction actually provides useful advantages for blood coagulation. Under the circumstances of vessel damage, the fibrinogen molecules directly treated by non-thermal plasma aggregate into polymers to assist blood coagulation while other components in the blood not directly in contact with plasma radiation would suffer negligible damage. As such, the phenomenon and mechanism reported herein can be promisingly useful in the non-thermal plasma-assisted blood coagulation or related medical treatments in the near future.

## Methods

### Reagents

Human fibrinogen, hemoglobin, γ-globulin, hematin (purity higher than 95%), and loading buffer were obtained from Sangon Biotech (Shanghai) Co., Ltd. Human serum albumin (HSA) was purchased from Sigma-Aldrich. Sodium chloride (NaCl) was purchased from Sinopharm Chemical Reagent Co., Ltd.). *N*-acetylimidazole and L, L-dityrosine dihydrochloride were purchased from J&K Scientific Ltd. All chemicals were used without further purification. Unless otherwise indicated, all fibrinogen solutions were obtained by dissolving an appropriate quantity of lyophilized fibrinogen powder in 0.9% NaCl solution. Hematin stock solution at 5 × 10^−3^ M was prepared prior to every experiment by dissolving certain amount hematin in NaOH (0.05 M) solution thoroughly. The concentration of hematin was estimated with the molar extinction coefficient as ε(398) = (12.2 ± 3) × 10^4^ M^−1^ cm^−1^ of monomeric state^35^. Fresh pig blood was taken from slaughterhouse and used for experiments immediately. Blood plasma was obtained after centrifuging at 8000 rmp for 5 min.

### The non-thermal plasma device and plasma treatment

In our study, a plasma jet device was used to generate the non-thermal plasma plume and the schematic diagram is shown in Supplementary Fig. 8. The high voltage stainless steel electrode was inserted into a quartz tube with one end closed which was inserted into a hollow barrel pipet. The output voltage was about 9.6 kV, the frequency was 9.27 kHz detected by oscillograph and the helium flow rate was 1 L/min. For blood and blood plasma sample, 270 μL of sample was mixed with 30 μL of hematin solution to obtain the desired hematin concentration and then the mixture was deposited into the cap of 1.5 mL centrifugal tube and exposed to non-thermal plasma treatment. Similarly for fibrinogen sample, certain amount of fibrinogen solution was mixed with diluted hematin solution and then 0.5 mL of the mixture was deposited into a 1.5 mL centrifugal tube or 0.3 mL of the mixture was deposited into the cap of the tube and exposed to non-thermal plasma treatment. The distance between the jet outlet and the solution surface was always kept at about 5 mm.

### Reducing sodium dodecyl sulfate-polyacrylamide gel electrophoresis (SDS-PAGE) analysis and fluorescence spectroscopy measurements

For the reducing SDS-PAGE analysis of the layer formed on blood, blood plasma, and fibrinogen solution surface after non-thermal plasma exposure, the layer was picked and dissolved in certain volume of 6 M urea (100 μL for the blood and blood plasma sample, and 50 μL for the fibrinogen sample). Then, 40 μL dissolved solution was mixed with 10 μL reducing loading buffer (5×, containing 250 mmol/L Tris-HCl (pH 6.8), 10% SDS, 0.5% Bromophenol blue, 50% glycerol, and 7.5% dithiothreitol (DTT)), vortexed and heated in boiling water for 10 min, and then separated by SDS-PAGE. The gels were stained with Coomassie Brilliant Blue R250. For fibrinogen solution at 2 mg/mL, a forty microliter sample solution was directly mixed with 10 μL reducing loading buffer and separated by SDS-PAGE under the same conditions as described above.

For steady state fluorescence measurements, the layer formed on fibrinogen solution (10 mg/mL) surface in the presence of hematin (3 × 10^−5^ M) after non-thermal plasma exposure for 2 min was firstly dissolved in 270 μL urea (6 M) with addition of 30 μL phosphate buffer (100 mM, pH = 7.2) and then measured on a fluorescence spectrometer (VARIAN Cary Eclipse). For the fibrinogen solution at 2 mg/mL, the fluorescence measurements were directly conducted. The scans were performed with quartz cuvettes with 1 mm optical path. Excitation wavelength was selected at 330 nm and both excitation and emission slit were 10 nm.

### Analysis of dityrosine in the membrane-like layer by UPLC

For UPLC analysis, the layer formed on fibrinogen solution (10 mg/mL) surface in the presence of hematin (3 × 10^−5^ M) after non-thermal plasma exposure for 2 min was hydrolyzed in 4 mL HCl (6 M) at 110 °C in nitrogen atmosphere overnight and then separated by a Waters UPLC (Ultra Performance Liquid Chromatography) system (ACQUITY UPLC H-Class) using an Waters BEH C18 column (2.1 × 50 mm). Analysis used a flow rate of 0.4 mL/min for with an isocratic solvent system (8% acetonitrile containing 0.1% TFA). Fluorescence detector (λ_ex_, 310 nm; λ_em_, 410 nm) was used for detecting dityrosine.

### UV-visible absorbance and Raman spectroscopy measurements of hematin with and without fibrinogen

UV–Vis absorption spectra of hematin (2 × 10^−5^ M) in the absence or presence of fibrinogen (5 mg/mL) were obtained by a UV–Vis spectrometer (SHIMADZH UV-2550) at ambient temperature. The scans were performed with quartz cuvettes with 1 mm optical path. For Raman measurements, a volume of 10 μL hematin solution (2 × 10^−5^ M) in the absence or presence of fibrinogen (5 mg/mL) was deposited onto quartz substrate and allowed to dry at 30 °C and the spectra were acquired with a HORIBA JOBIN YVON XploRA spectrometer which worked in the confocal mode. The spectra were collected on the edge of the “coffee-ring” arising from the outward flow of solvent^36^. A 100× objective (Olympus, MPlanN, NA 0.9) was used to focus the laser onto the sample (with spot size ∼3 μm in diameter) and also collect the back-scattered Raman light into the detector. The laser power at the sample was reduced to approximately 5.0 mW. All the spectra represent the average from three cycles of 60 seconds Raman spectra acquired consecutively under the same conditions. For every sample, Raman measurements were conducted at 5 points at least, and at every point, the Raman spectra were collected with the parameters as described above.

## Additional Information

**How to cite this article**: Ke, Z. and Huang, Q. Haem-assisted dityrosine-cross-linking of fibrinogen under non-thermal plasma exposure: one important mechanism of facilitated blood coagulation. *Sci. Rep.* **6**, 26982; doi: 10.1038/srep26982 (2016).

## Supplementary Material

Supplementary Information
