# Supplementary material for: Haem-assisted dityrosine-cross-linking of fibrinogen under non-thermal plasma exposure: one important mechanism of facilitated blood coagulation
Source: Sci Rep. 2016 May 27;6:26982. doi: 10.1038/srep26982 (PMC4882584; doi:10.1038/srep26982)
Supplement: Supplementary Information [file srep26982-s1.doc]

**Supplementary Information**

**Haem-assisted dityrosine-cross-linking of fibrinogen under non-thermal plasma exposure: one important mechanism of facilitated blood coagulation**

Zhigang Ke ^1^, Qing Huang ^1, 2^

*^1.^Key Laboratory of Ion Beam Bioengineering, Hefei Institutes of Physical Science, Chinese Academy of Sciences, Hefei 230031, China*

*^2^. National Synchrotron Radiation Laboratory, University of Science & Technology of China, Hefei 230026, China*

*Correspondence and requests for materials should be addressed to Q.H. (email: huangq@ipp.ac.cn)*


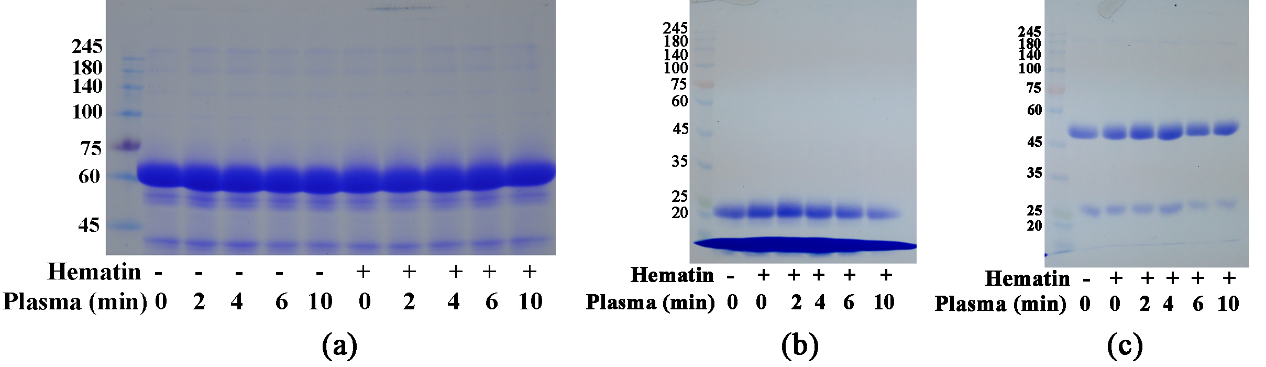


**Supplementary Figure 1. Hematin cannot assist the cross-linking of human serum albumin (HSA, a), hemoglobin (b), and γ-globulin (c) under non-thermal plasma exposure.** Reducing SDS-PAGE analysis of HSA solution (2 mg/mL), hemoglobin (10 mg/mL), and γ-globulin (2 mg/mL) in the absence or presence of hematin (5×10^-6^ M) with non-thermal plasma exposure for different time.


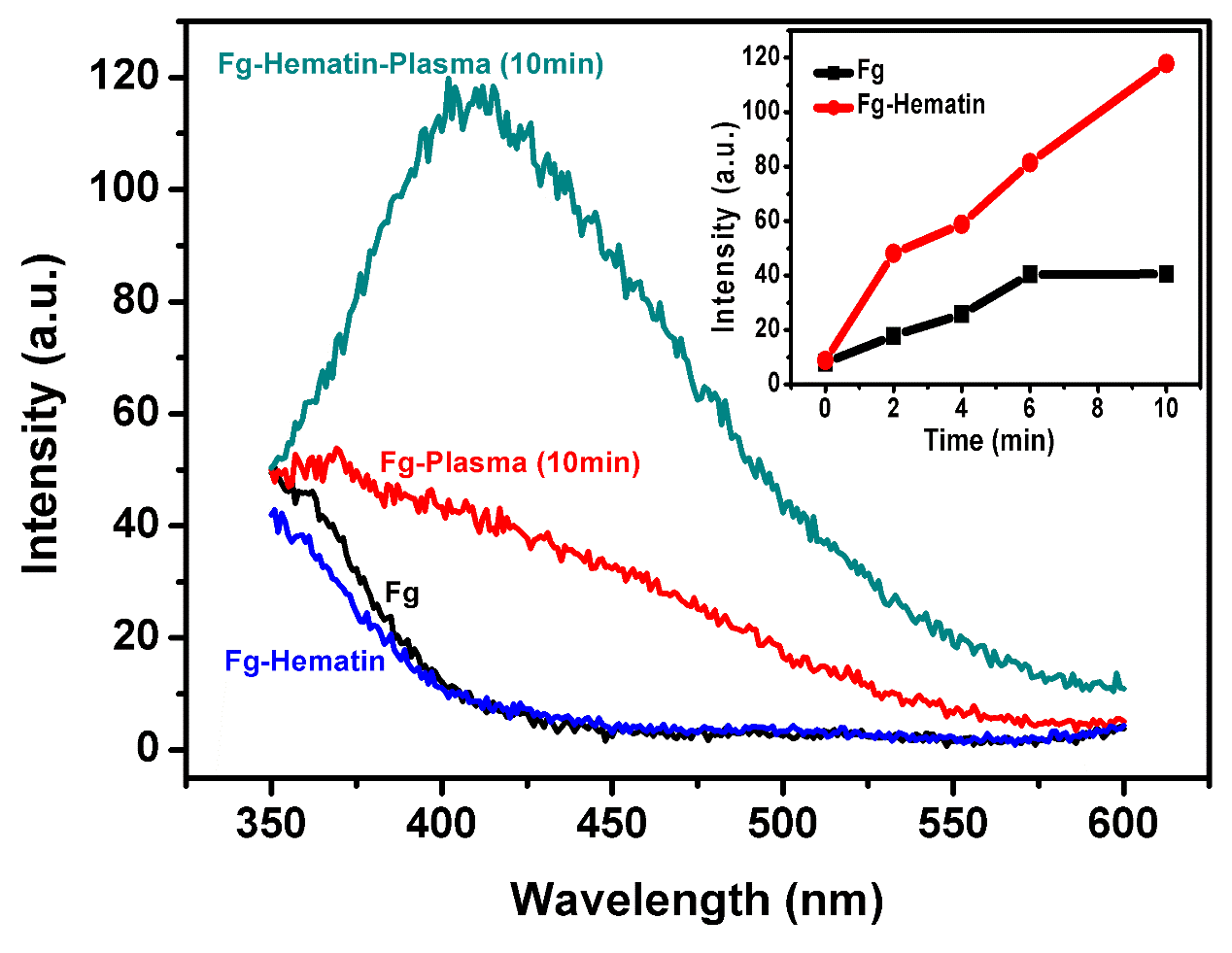


**Supplementary Figure 2. Dityrosine formation in fibrinogen solution in the presence of hematin after non-thermal plasma treatment.** Fluorescence spectra of fibrinogen solution (2 mg/mL) in the absence or presence of hematin (5×10^-6^ M) with non-thermal plasma exposure for different times. Excitation wavelength: 330 nm; Excitation and emission slit: 10 nm.


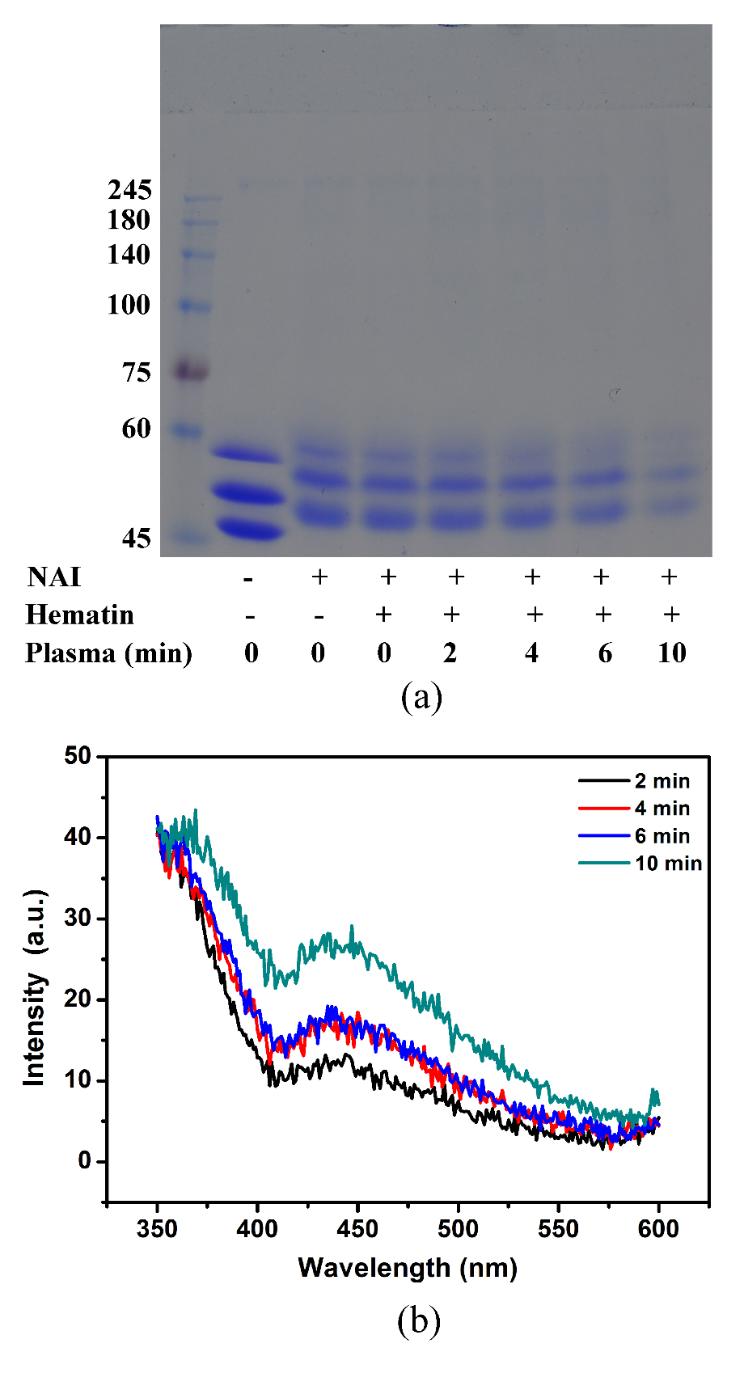


**Supplementary Figure 3. Tyrosyl residues modification inhibited fibrinogen cross-linking and fluorescence band formation.** (**a**) Reducing SDS-PAGE and (**b**) fluorescence spectra analysis of modified fibrinogen by *N*-acetylimidazole after exposure to non-thermal plasma in the presence of hematin (5×10^-6^ M). 2 mL fibrinogen solution at 5 mg/mL in 0.01 M Tris-HCl buffer (pH7.5) was added with certain amount of *N*-acetylimidazole to achieve final concentration at 0.15 M. The reaction mixture was placed at room temperature for 1 hour and then dialyzed against 0.9% NaCl solution overnight. The obtained modified fibrinogen sample was exposed to non-thermal plasma treatment and analyzed with SDS-PAGE and fluorescence measurements.


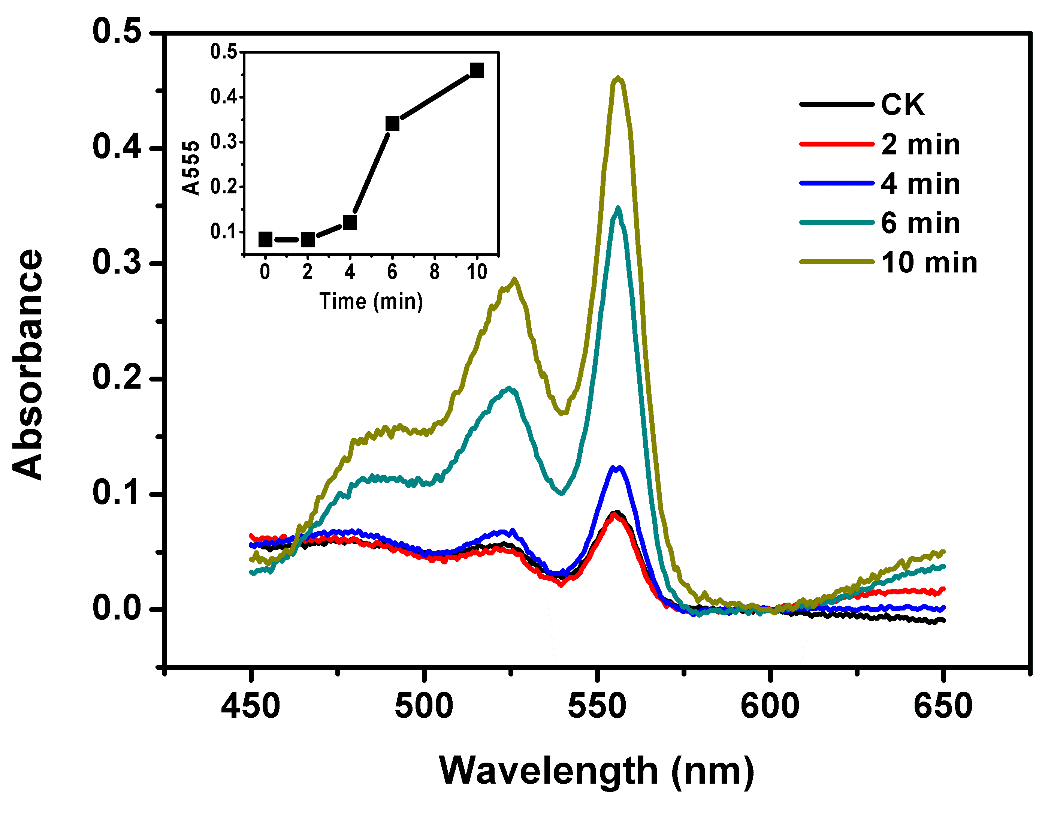


**Supplementary Figure 4. Non-thermal plasma exposure increased free haem concentration in pig blood.** UV-Vis absorbance spectral measurement of free heme by the pyridine hemochromogen assay in the pig blood sample after non-thermal plasma treatment ([*Blood*](http://www.ncbi.nlm.nih.gov/pubmed/11535514) 2001, 98, 1802-1811). 500 µL blood sample was treatment by non-thermal plasma for different time and centrifuged at 5000 rmp for 5 min. 0.3 mL solution A (3 parts pyridine and 1 part 1 M NaOH) was added to 150 µL of the obtained supernatant, mixed thoroughly, and used as the baseline. Next, 1-2 mg sodium dithionite was added and the absorbance spectrum between 450 and 600 nm was recorded.


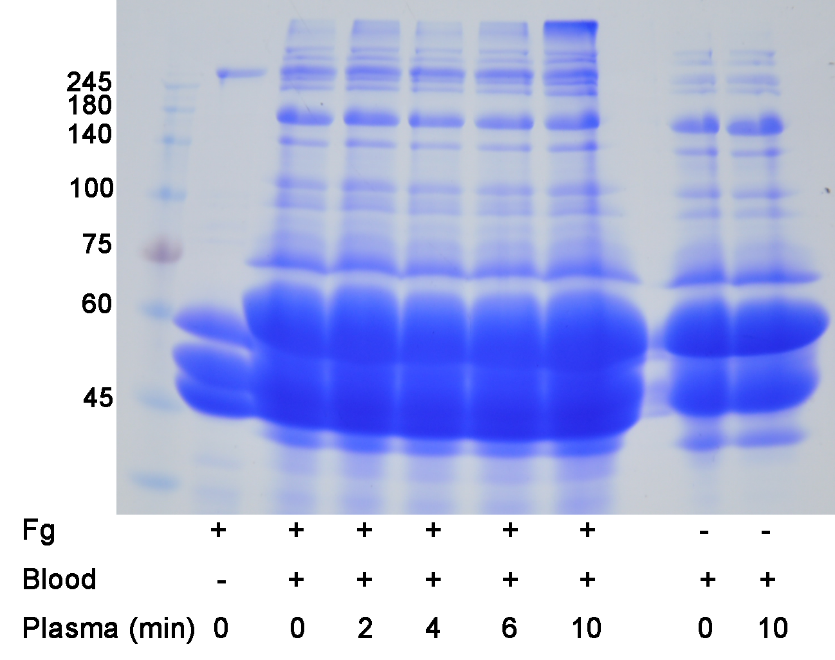


**Supplementary Figure 5. Blood assisted fibrinogen cross-linking under non-thermal plasma exposure.** Reducing SDS-PAGE analysis of fibrinogen in the presence of blood after non-thermal plasma treatment for different time. 0.45 mL fibrinogen solution (2 mg/mL in 0.9% NaCl) was mixed with 50 µL of pig blood and then the sample was exposed to non-thermal plasma treatment. Immediately after non-thermal plasma treatment, the sample was separated by SDS-PAGE. 0.45 mL NaCl (0.9%) mixed with 50 µL of pig blood was used as the control.


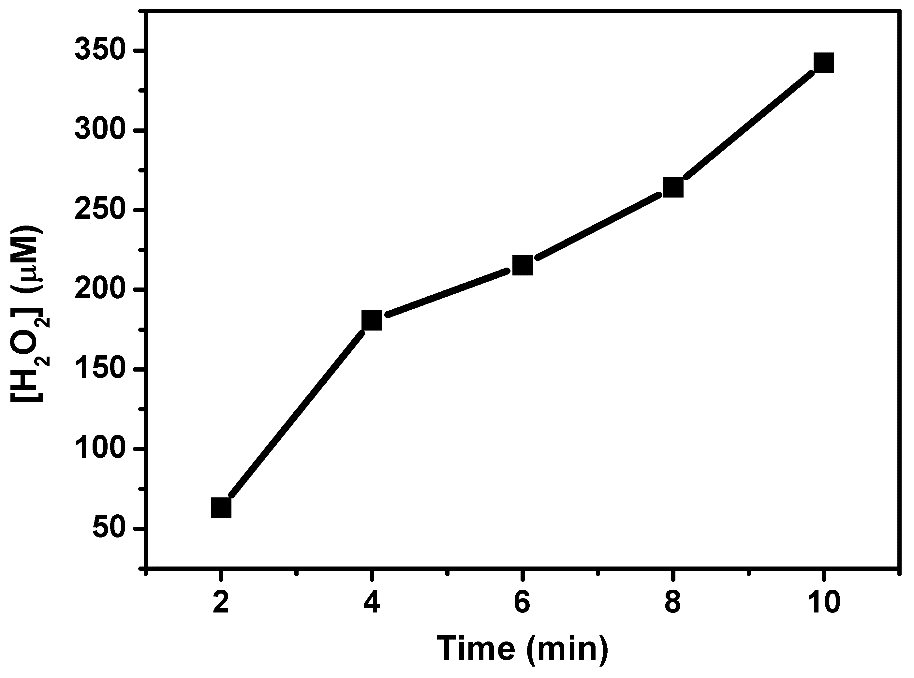


**Supplementary Figure 6. Hydrogen peroxide production in solution by non-thermal plasma exposure.** Hydrogen peroxide concentration in 0.9% NaCl after non-thermal plasma treatment for different time was determined spectrophotometrically at 410 nm after mixing with titanium sulfate in acidic condition (*Ind. Eng. Chem. Anal. Ed.* 1943, 15, 327-327). 0.5 mL NaCl (0.9%) solution was exposed to non-thermal plasma treatment for different time and then 0.4 mL of plasma-treated sample was added to 0.6 mL of detection reagent (containing 1.5 M H_2_SO_4_ and 0.15 mM titanium sulfate). The reaction was allowed to proceed for 30 min at room temperature and then 2 mL of distilled water was added to the reaction mixture. Absorbance at 407 nm of the mixture was recorded.


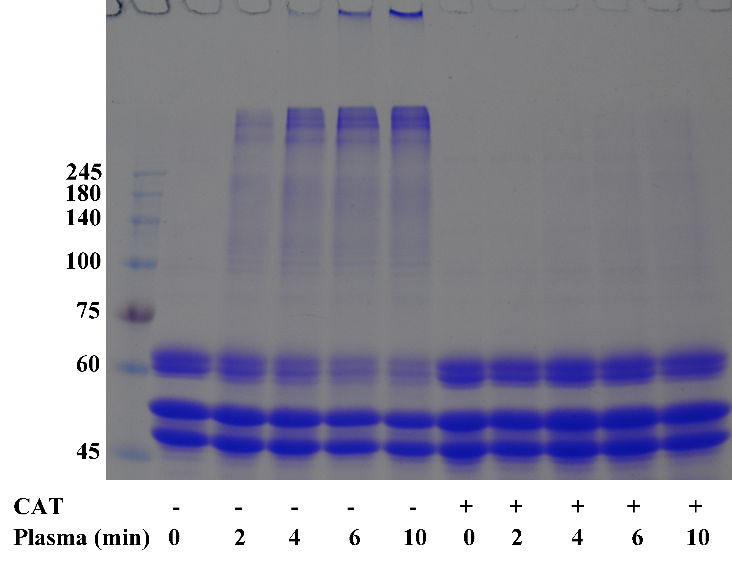


**Supplementary Figure 7. Catalase (CAT) inhibited fibrinogen cross-linking assisted by hematin under non-thermal plasma exposure.** 0.5 mL fibrinogen (2 mg/mL) and hematin (5×10^-6^ M) mixture in the absence or presence of catalase (0.2 mg/mL) was exposed to non-thermal plasma treatment for different times and then the sample were separated by reducing SDS-PAGE.


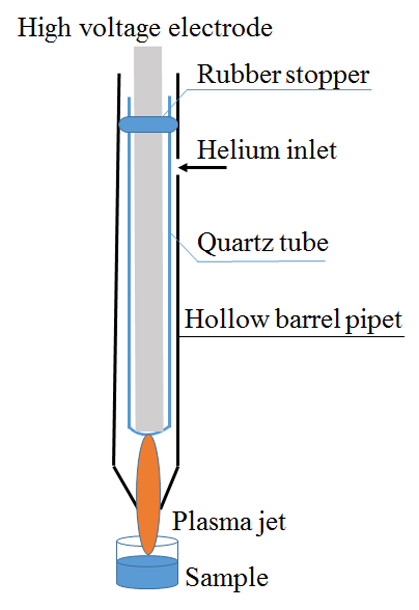


**Supplementary** **Figure 8. Schematic diagram of non-thermal plasma jet device.**

**References**

# Waqener, F. A. et al. Heme is a potent inducer of inflammation in mice and is counteracted by heme oxygenase. *Blood* 98, 1802-1811 (2001)

# Eisenberg, G. M. Colorimetric determination of hydrogen peroxide. *Ind. Eng. Chem. Anal. Ed.* 15, 327–328 (1943)

# 
